# Aneurismal Tumor of the Right Alveolar Process and Vault of the Mouth Treated by Injection

**Published:** 1891-08

**Authors:** John S. Marshall

**Affiliations:** Chicago, Ill.


ARTICLE IV.

### ANEURISMAL TUMOR OF THE RIGHT ALVEOLAR PROCESS AND VAULT OF THE MOUTH TREATED BY INJECTION.

BY JOHN S. MARSHALL, M. D., CHICAGO, ILL.

Read before the New Jersey State Dental Society at the annual meeting, July, 1890.

Mr. C. B. H—, of Chicago, American, aged twenty-six years, occupation, travelling salesman, was referred to me, December 26, 1889, for counsel and treatment, by Dr. M. Stout, of Chicago, with the following history: Some eighteen or twenty months previous to this examination the gentleman had submitted to the extraction of all his superior teeth except the central incisiors. The operation was performed under nitrous oxide gas; the mouth was badly bruised and lacerated on account of the difficulty in extracting the teeth. A few weeks afterwards he noticed a swelling upon the inner side of the right alveolar ridge, which continued to enlarge as the months went by, and prevented the making of the artificial denture, which he was anxious to have placed in his mouth. There was no pain or uncomfortable feeling about the tumor, except when engaged in vigorous exercise; at such times pulsation in the part would become very marked and disagreeable.

According to his own statement, he had "consulted several dentists in relation to the character of the swelling: some did not know what it was; one said it was an accumulation of pus, another that it was a 'watery tumor,' and a third that it was 'wind,' and asked the privilege of letting it out." This very kind offer, however, was declined, much to the permanent benefit and longevity of our patient.

Examination of the mouth revealved the superior teeth all gone except the central incisors, and a pulsating tumor about one and one-half inches in length, by one inch in width, egg shaped in form, with the small end pointing forward, and occupying the right side of the vault of the mouth, from the outer wall of the alveolar process to the median line, and from the tuberosity of the maxilla forward to a line drawn through the cuspid region.

In character it was soft, fluctuating, compressible, and with very marked pulsation. In color it was slightly deeper in tint than the surrounding mucous membrane. Upon puncturing it with an exploring needle, a jet of arterial blood followed its withdrawal, and continued to spurt for about half a minute, when the hemorrhage ceased.

The diagnosis was aneurismal tumor of the posterior palatine artery, with possible anastomosis with some branch of the superior maxillary artery, the result of injury in the extraction of the teeth. An operation was advised, and the gentleman agreed to report in about two months, Business engagements prevented his keeping this appointment, and when he next called to arrange for the operation I was out of town on my summer vacation. He was also on vacation, and could not await my return, and therefore sought other advice.

October 10, 1889, I saw him again, at which time he gave me the following additional history: That in July he had been operated upon for the removal of the tumor and had nearly died under the operation from hemorrhage, and was afterwards confined to his bed for two months with blood-poisoning.

The cast which I now show you is a copy of his upper jaw, taken on December 17, 1889, a few days before I operated on him. You will see by this that there was no material improvement in the condition as described in the examination made a year previously.

The surgical treatment of aneurisms, as you all know, is by ligating the artery near the cardiac or distal extremity of the sac, or both; by compression, either instrumental or digital; by the introduction of foreign substances into the sac, like cat-gut, horse-hair, or fine iron or silver wire; by manipulation, by acupuncture, by galvano-puncture, by the injection of coagulating fluids, and in the case of small anastomosing or cirsoid aneurisms by dissection,—the particular method adopted being controlled by the character and location of the aneurisms, the chances of danger to life, the possibilities of a cure, and the individual preferences of the operator. In all such cases as are susceptible of ligation, this is by far the most satisfactory surgical procedure. But in those which from their location cannot be reached by this method some one of the other means may be employed.

In the case under consideration treatment by ligation, compression, manipulation, acupuncture, and dissection was out of the question; the means at our command were therefore limited to three methods: the introduction of foreign substances, like wire, etc., galvano-puncture, and injection.

Treatment by the introduction of foreign substances, either animal or metallic, seemed slow and unsatisfactory. and gave little hope of success, for I had been unable to find a single case on record of a cure by this means, while the dangers from embolism were great.

Galvano-puncture was considered too tedious an operation, from the fact that several would most likely be required to effect a cure, while the dangers from embolism and from hemorrhage as a result of sloughing at the points of puncture made it seem extremely hazardous. I, therefore, decided to treat the case by injection, though this method is by no means free from the dangers already enumerated. The injection method in aneurisms of large arteries is generally considered positively unsafe; and in those occurring in terminal branches of arteries it has not met with much favor by the profession, chiefly for the reason that several fatal results from embolism were recorded soon after its introduction, and thus deterred many from giving it a trial in those cases which might be considered favorable.

The danger of this method in aneurisms connected with small arteries, it seems to me, is not in the method itself, but in the kind and strength of the coagulating fluids used.

The agents which have been suggested are numerous, among which are acetate of lead, acetic acid, iodine, ergotine, and the perchloride of iron. The perchloride has generally been given the preference, used in small quantities and weak solutions of one to two per cent.

The injections of solutions in the above quantity and strength produce very slow coagulation, and when the clot is formed it is soft and friable; as a consequence, it is easily broken up and floated away, giving rise to embolism in remote parts of the circulating system, with all its accompanying dangers. The dangers in acupuncture. galvano-puncture, and the introduction of foreign substances into the sac are for the same reasons equally great.

The perchloride of iron is a vigorous coagulant, and quite escharotic and antiseptic when used in full strength. A one or two-per-cent. solution is very mild in its styptic and coagulant qualities, is not escharotic, and has no value as an antiseptic.

What is needed in the treatment of this class of cases is to produce a firm clot, instantaneously if possible, and to maintain it in an aseptic condition, without the dangers of sloughing or hemorrhage.

In the perchloride solution of proper strength it would seem that we had all these requirements.

By the production of instantaneous and complete coagulation of the blood in aneurisms of this class and nævi, the dangers from embolism in remote parts of the body would seem to be entirely overcome and thus one great objection to this method removed; at least this was my thought upon the matter, and I determined to try it in this case,in preference to the other methods which might have been chosen.

In order to produce complete and firm coagulum instantaneously, it would be necessary to use a solution of much greater strength than had been previously recommended.

From this size of the tumor I concluded that in all probability it contained from one to one and a half ounces of blood, and that the introduction of five minims of the following solution, perchloride of iron one part, water four parts, would not be sufficiently escharotic to do any mischief when diluted with this quantity of blood.

On December 22, 1889, I injected into the tumor five minims of the above solution, which produced instantaneous coagulation, making the tumor feel as firm as a fibroma; considerable pain followed the injection, but this gradually subsided after a few minutes, but he complained for some hours of a strange fullness of the right side of the head. On withdrawing the hypodermic needle a little oozing occurred, which immediately discolored the mucous membrane for a little distance around the puncture made by the needle. No other unpleasant symptoms followed.

December 28 the mucous membrane, at the point punctured by the needle, sloughed, leaving an opening into the sac about the size of a silver half-dime, exposing the hard clot. There was not the slightest hemorrhage, but considerable anxiety was felt for fear of such an occurrence.

Tiersch's antiseptic solution was constantly used as a mouth-wash during the whole progress of the case, and after the slough occurred the sac was syringed with the same solution at short intervals, day and night.

December 31 the clot was broken up and removed, when it was found that the aneursism also occupied the antrum of Highmore, and had produced absorption of the palatine process of the superior maxillary bone and the nasal wall of the antrum, leaving a large opening into the nasal fossa. The case progressed without a single draw-back from this time onward. The opening into the antrum and floor of the nasal fossa finally closed. There had been no recurrence and the patient considers himself perfectly well. Cast No. 2 shows his present condition.

*Remarks.*—The sloughing of the mucous membrane at the point of puncture proves that the strength of the solution was too great for absolute safety, and, should I be called upon to treat another case of this class, I should not feel warranted in using a solution stronger than one in six or eight parts of water. This, I think, would produce the desired results, without the dangers of causing a slough and possible hemorrhage.—*International Dent. Journal.*

---

